# A nomogram based on conventional ultrasound and elastography for diagnosing BI-RADS category 3–5 lesions

**DOI:** 10.1186/s12880-026-02216-3

**Published:** 2026-02-11

**Authors:** Yi Chen, Yongbin Li, Jieyu Zhong, Haiying Zhou, Yanping Chen, Yan Chen, Desheng Sun

**Affiliations:** 1 Department of Ultrasonography, Peking University Shenzhen Hospital, 1120 Lianhua Road, Shenzhen, Guangdong China; 2 Department of Ultrasonography, Shenzhen Children’s Hospital, Shenzhen, Guangdong China; 3 Department of Ultrasonography, Jiangmen Central Hospital, Jiangmen, Guangdong China

**Keywords:** Ultrasound, Elastography, BI-RADS, Nomogram

## Abstract

**Objective:**

To develop a nomogram using conventional ultrasound and elastography to offer a more objective and accurate Breast Imaging Reporting and Data System (BI-RADS) classification, and minimize unnecessary biopsies.

**Methods:**

We retrospectively analyzed 689 BI-RADS category 3–5 lesions diagnosed by ultrasound. The cases were assigned to a training group and a validation group based on the timing of the ultrasound examination. The nomogram was constructed using multivariate logistic regression analysis and evaluated through Receiver Operating Characteristic curves, calibration curves, and decision curve analysis. Conventional BI-RADS 4a lesions were re-evaluated using the nomogram.

**Results:**

The nomogram incorporated 10 predictors: age, indistinct margin, angular margin, microlobulated margin, spiculated margin, hyperechoic halo, orientation, calcification, vascularity, and elasticity assessment. The area under the curve values for the nomogram in the training and validation group were 0.906 (95% confidence interval [CI]: 0.882–0.931) and 0.921(95%CI: 0.854–0.988), respectively. The calibration curves demonstrated good agreement between the nomogram predictions and actual malignancy outcomes. The decision curve analysis indicated that the nomogram had good clinical applicability. In the training group, 13% (41/304) of the conventional BI-RADS 4a lesions were downgraded to BI-RADS 3 by the nomogram, with only one malignancy (1/41, 2%). In the validation group, 17% (7/42) of conventional BI-RADS 4a lesions were downgraded through the nomogram, with no malignancies (0/7, 0%).

**Conclusions:**

We developed a high-performing nomogram based on conventional ultrasound and elastography. The nomogram could provide individualized malignancy risk predictions for BI-RADS 3–5 lesions using fewer indicators, and help avoid unnecessary biopsies.

**Clinical trial number:**

Not applicable.

**Supplementary Information:**

The online version contains supplementary material available at 10.1186/s12880-026-02216-3.

## Introduction

According to the latest global cancer data from 2020, breast cancer has become the most common cancer across the world [1]. Breast cancer screening significantly enhances early diagnosis rates, survival rates, and quality of life [2, 3]. Ultrasonography, a crucial imaging screening method for breast cancer [4, 5], is commonly performed using the 5th edition of the Breast Imaging Reporting and Data System (BI-RADS) established by the American College of Radiology (ACR) [6]. In the BI-RADS classification system, the morphological features of breast lesions serve as the core basis for classification. Color Doppler and elastography are used only as supplementary features and cannot be independently applied for classification, likely due to their operator dependence and variability in equipment parameters. However, studies have confirmed that the addition of color Doppler and elastography to B-mode helps improve the efficiency of differentiating benign and malignant breast lesions and reduce unnecessary biopsies [7–9]. However, this system faces several challenges in clinical application: (1) Subjective classification: The BI-RADS classification of lesions is influenced by the subjective judgment and experience of sonographers, which increases the risk of missed diagnosis and misdiagnosis of breast cancer to a certain extent [10]. (2) Broad malignancy range: The ACR BI-RADS guidelines recommend biopsy for all BI-RADS category 4 lesions [6]. However, BI-RADS category 4 encompasses a wide range of malignancy probabilities (2%–95%), resulting in many benign lesions undergoing unnecessary biopsies, particularly BI-RADS 4a lesions (2%–10%) [11]. This exercise not only wastes medical resources but also heightens the psychological burden on patients.

To address these issues, various studies have attempted to develop risk prediction models to enhance the diagnostic performance of ultrasound in distinguishing between benign and malignant breast lesions as well as to refine the BI-RADS system. Among these, the nomogram stands out as a widely used risk assessment tool in oncology [12]. Nomograms incorporate and illustrate important predictive factors, and transform complex statistical models into simple, intuitive visual graphs, which provides individualized probabilities for clinical events, aligns with the needs of personalized medicine and offers high predictive efficiency and clinical utility [13, 14].

Thus, we herein aimed to develop and validate a nomogram based on conventional ultrasound and elastography to provide individualized malignancy risk predictions and achieve more objective and accurate BI-RADS classification for BI-RADS category 3–5 lesions. By doing so, we seek to minimize unnecessary biopsies.

## Materials and methods

### Patients

This study received approval to waive informed consent from the Ethics Committee of Peking University Shenzhen Hospital (Approval number: 2022135). We retrospectively collected the ultrasound images and clinicopathological data of BI-RADS category 3–5 lesions diagnosed via ultrasound and histopathological examination at Shenzhen Hospital of Peking University from January 2019 to July 2021. The exclusion criteria comprised: (1) incomplete clinical data, (2) unclear pathological findings of breast lesions, and (3) incomplete or unsatisfactory quality of ultrasound images. The patients were categorized into a training group (January 2019 to December 2020) and a validation group (January 2021 to July 2021) based on the timing of their breast ultrasound examination.

### Ultrasonography

The US examinations (grayscale ultrasound, color Doppler ultrasound and strain elastography) were performed by examiners with over 5 years of breast ultrasound experience, prior to biopsy or surgery. High-resolution ultrasound systems (Mindray Resona 7, Mindray Resona 8, Canon Aplio 500, etc.) equipped with high-frequency linear array probes (> 10 MHz) were used. Color Doppler ultrasound examination: (1) Parameters including pulse repetition frequency (PRF), wall filter (WF), depth penetration and so on were flexibly adjusted according to the blood flow intensity of the lesions. (2) The probe should be placed gently without compression to prevent small blood vessels from being compressed and compromising blood flow visualization. Strain elastography examination: (1) Upon identification of the breast lesion on grayscale ultrasound (avoiding calcification, cystic/necrotic zones, and major vessels), elastography mode was activated. (2) A region of interest was defined, encompassing the subcutaneous fat superiorly and the pectoral muscle inferiorly, and extending more than 5 mm beyond the lesion laterally. (3) Tissue stiffness was displayed on a color scale (red=hard, green=intermediate, blue=soft; customizable). (4) The probe was kept perpendicular to the skin at all times with gentle and steady manual compression. (5) Elastograms were acquired when the “spring” icon on the screen was stably displayed in green.

### Ultrasound image interpretation

Each breast lesion’s ultrasound images included at least two planimetric views of gray-scale images (longitudinal and transverse planes of the lesion), color Doppler images, and elastographic images. Examiners interpreted all ultrasound images, while possessing knowledge of patients’ ages but blinded to the pathological results of the lesions. The interpretations were performed in a fixed sequence (grayscale images first, then elastographic and color Doppler images) and based on the 5th edition of the ACR BI-RADS guideline [6] and the elasticity scoring criteria of Itoh et al. [15]. Examiners described the ultrasound characteristics and assigned BI-RADS categories to each lesion. The training group involved two examiners with 10 years of experience in breast ultrasound in interpreting images (examiners A and B). Any disagreements were resolved through discussions with a senior examiner with over 20 years of experience (examiner C). In the validation group, the interpretations were conducted by an examiner with 10 years of experience (examiner D). Characteristics such as age and ultrasound features were recorded (Table 1). Specifically, microcalcification was noted when both macrocalcification and microcalcification were present in the lesion, and internal blood supply was indicated when both edge blood supply and internal blood supply were observed. In the ACR BI-RADS guideline, elasticity assessment is categorized into soft, medium, and hard grades. With reference to the ACR BI-RADS guideline and the Itoh scoring system, elasticity scores of 1–2 are defined as soft, a score of 3 as medium, and scores of 4–5 as hard [6, 15].

**Table 1:** The age and ultrasound characteristics

Characteristics	Subcategory
Age	<40y; ≥40y
Shape	Regular; Irregular
Circumscribed margin	Yes; No
Indistinct margin	Yes; No
Angular margin	Yes; No
Microlobulated margin	Yes; No
Spiculated margin	Yes; No
Hyperechoic halo	Yes; No
Orientation	Parallel; Not parallel
Echo pattern	Hypoechoic; Isoechoic/Hyperechoic/Heterogeneous; Complex cystic and solid
Posterior features	Shadowing; No posterior features; Enhancement; Combined pattern
Calcification	Absent; Macrocalcification; Microcalcification
Vascularity	Absent; Edge blood supply; Internal blood supply
Elasticity assessment	Soft; Medium; Hard

### Pathological result

Breast lesions were classified as benign or malignant based on the final pathological results. Further analysis of specific pathological subtypes was not performed in this study.

### Statistical analysis

Data were analyzed using SPSS (IBM SPSS Statistics, version 25.0) and R software (version 3.6.1). Categorical variables are presented as frequencies and percentages. Two-sided statistical significance was employed, with *P* < 0.05 considered to indicate statistical significance. Kappa consistency test was used to evaluate the inter-observer agreement of ultrasound characteristics and BI-RADS classification. Univariate and multivariate logistic regression analyses were performed to screen the predictors. Multicollinearity Test was performed to exclude model distortion due to the high correlation between predictors. Using the selected predictors as variables, the prediction model and nomogram were constructed based on multivariate logistic regression analysis (enter). The nomogram was assessed using Receiver Operating Characteristic (ROC) curves, calibration curves, and decision curve analysis (DCA) [16]. Internal validation was performed using the bootstrap resampling method, while external validation was conducted in the validation group. Using the constructed nomogram to re-predict the malignancy probability for lesions empirically classified as BI-RADS 4a, and based on the ACR BI-RADS guideline, which states the malignancy probability for BI-RADS category 3 lesions is 0% < probability ≤ 2% [6], lesions with a nomogram-predicted malignancy probability ≤ 2% are therefore downgraded to BI-RADS category 3.

## Results

### Characteristics of patients and lesions

A total of 689 breast lesions were included in this study, with 615 cases (396 benign lesions, 219 malignant lesions) in the training group and 74 cases (58 benign lesions, 16 malignant lesions) in the validation group. The final dataset contained no missing values. The age and ultrasound characteristics of the lesions are shown in Table 2. Table 3 presents the results of inter-observer agreement for ultrasound characteristics and BI-RADS classification in the training group.

**Table 2:** Age and ultrasound characteristics of lesions

Characteristics		Training group(*N*%)	Validation group(*N*%)
Age	<40	229(37.2)	36(48.6)
	≥ 40	386(62.8)	38(51.4)
Shape	Regular	118(19.2)	7(9.5)
	Irregular	497(80.8)	67(90.5)
Circumscribed margin	Yes	142(23.1)	15(20.3)
	No	473(76.9)	59(79.7)
Indistinct margin	No	420(68.3)	52(70.3)
	Yes	195(31.7)	22(29.7)
Angular margin	No	311(50.6)	40(54.1)
	Yes	304(49.4)	34(45.9)
Microlobulated margin	No	323(52.5)	73(98.6)
	Yes	292(47.5)	1(1.4)
Spiculated margin	No	537(87.3)	67(90.5)
	Yes	78(12.7)	7(9.5)
Hyperechoic halo	No	572(93.0)	73(98.6)
	Yes	43(7.0)	1(1.4)
Orientation	Parallel	510(82.9)	59(79.7)
	Not parallel	105(17.1)	15(20.3)
Echo pattern	Hypoechoic	551(89.6)	66(89.2)
	Isoechoic/Hyperechoic/Heterogeneous	27(4.4)	3(4.1)
	Complex cystic and solid	37(6.0)	5(6.8)
Posterior features	Shadowing	88(14.3)	9(12.2)
	No posterior features	372(60.5)	50(67.6)
	Enhancement	112(18.2)	3(4.1)
	Combined pattern	43(7.0)	12(16.2)
Calcification	Absent	393(63.9)	39(52.7)
	Macrocalcification	35(5.7)	4(5.4)
	Microcalcification	187(30.4)	31(41.9)
Vascularity	Absent	289(47.0)	50(67.6)
	Edge blood supply	132(21.5)	8(10.8)
	Internal blood supply	194(31.5)	16(21.6)
Elasticity assessment	Soft	194(31.5)	12(16.2)
	Medium	258(42.0)	36(48.6)
	Hard	163(26.5)	26(35.1)
BI-RADS category	3	39(6.3)	4(5.4)
	4a	304(49.4)	42(56.8)
	4b	90(14.6)	14(18.9)
	4c	84(13.7)	8(10.8)
	5	98(15.9)	6(8.1)
Total		615(100)	74(100)

**Table 3:** Inter-observer agreement for ultrasound characteristics and BI-RADS classification

Characteristics	Kappa value
Shape	0.35
Circumscribed margin	0.34
Indistinct margin	0.28
Angular margin	0.25
Microlobulated margin	0.26
Spiculated margin	0.32
Hyperechoic halo	0.42
Orientation	0.38
Echo pattern	0.42
Posterior features	0.51
Calcification	0.70
Vascularity	0.65
Elasticity assessment	0.55
BI-RADS classification	0.40

### Screening of predictors

In the training group, Table 4 shows the ability of predictors to differentiate malignant from benign lesions. Hyperechoic halo and non-parallel orientation exhibited low sensitivity, but high specificity in differentiating between benign and malignant lesions. In the training group, univariate logistic regression analysis revealed that only the echo pattern lacked statistical significance in differentiating between malignant and benign breast lesions (*P* > 0.05) (Table 5). The predictors, excluding echo patterns, were examined for multicollinearity, with all variance inflation factors being less than 5, indicating no multicollinearity (Annexed Table 1). Therefore, the predictors, excluding echo pattern, were entered into the multivariate logistic regression analysis (stepwise), which showed statistically significant differences in age, indistinct margin, angular margin, microlobulated margin, spiculated margin, calcification, vascularity, and elasticity assessment (*P* < 0.05) (Table 5).

**Table 4:** Diagnostic efficacy of predictors in the training group

Predictors	Sensitivity	Specificity	Accuracy	Youden index	PPV	NPV
≥ 40y	80.40%	47.00%	58.90%	0.27	45.60%	81.20%
Irregular shape	95.00%	27.00%	51.20%	0.22	41.90%	90.70%
Not circumscribed margin	94.50%	32.80%	54.80%	0.27	43.80%	91.50%
Indistinct margin	53.90%	80.60%	71.10%	0.34	60.50%	76.00%
Angular margin	64.40%	58.80%	60.80%	0.23	46.40%	74.90%
Microlobulated margin	69.90%	64.90%	66.70%	0.35	52.40%	79.60%
Spiculated margin	28.80%	96.20%	72.20%	0.25	80.80%	70.90%
Hyperechoic halo	17.80%	99.00%	70.10%	0.17	90.70%	68.50%
Not parallel orientation	27.90%	88.90%	67.20%	0.17	58.10%	69.00%
Hypoechoic	92.20%	11.90%	40.50%	0.04	36.70%	73.40%
Shadowing posterior features	20.10%	88.90%	64.40%	0.09	50.00%	66.80%
Microcalcification	52.50%	81.80%	71.40%	0.34	61.50%	75.70%
Edge and internal blood supply	80.80%	62.40%	68.90%	0.43	54.30%	85.50%
Medium and hard elasticity	89.00%	42.90%	59.30%	0.32	46.30%	87.60%

^*^ PPV: positive predictive value; NPV: negative predictive value

**Table 5:** Univariate and multivariate logistic regression analysis in the training group

Predictors	Univariate analysis		Multivariate analysis	
	OR (95% CI)	*P*	OR (95% CI)	*P*
Age				
<40	Reference		Reference	
≥ 40	3.63(2.46–5.34)	0	5.03(2.92–8.66)	0
Shape				
Regular	Reference		Reference	
Irregular	7.00(3.67–13.35)	0	1.25(0.43–3.60)	0.681
Circumscribed margin				
Yes	Reference		Reference	
No	8.43(4.54–15.65)	0	0.71(0.23–2.24)	0.562
Indistinct margin				
No	Reference		Reference	
Yes	4.84(3.36–6.97)	0	3.68(2.00-6.77)	0
Angular margin				
No	Reference		Reference	
Yes	2.58(1.84–3.64)	0	2.071(1.16–3.69)	0.013
Microlobulated margin				
No	Reference		Reference	
Yes	4.29(3.01–6.11)	0	1.97(1.13–3.42)	0.017
Spiculated margin				
No	Reference		Reference	
Yes	10.26(5.67–18.56)	0	3.78(1.76–8.11)	0.001
Hyperechoic halo				
No	Reference		Reference	
Yes	21.23(7.48–60.32)	0	2.81(0.72–10.97)	0.138
Orientation				
Parallel	Reference		Reference	
Not parallel	3.09(2.01–4.75)	0	1.81(0.94–3.48)	0.078
Echo pattern		0.283		
Hypoechoic	Reference			
Isoechoic/Hyperechoic/Heterogeneous	0.61(0.25–1.46)	0.261		
Complex cystic and solid	0.64(0.30–1.35)	0.241		
Posterior features		0		0.094
Shadowing	Reference		Reference	
No posterior features	0.39(0.25–0.63)	0	0.93(0.45–1.95)	0.853
Enhancement	0.72(0.41–1.27)	0.26	2.07(0.84–5.10)	0.115
Combined pattern	1.15(0.55–2.39)	0.708	0.81(0.27–2.41)	0.703
Calcification		0		0.001
Absent	Reference		Reference	
Macrocalcification	0.50(0.19–1.31)	0.157	0.58(0.17–1.96)	0.38
Microcalcification	4.74(3.27–6.88)	0	2.54(1.51–4.24)	0
Vascularity		0		0
Absent	Reference		Reference	
Edge blood supply	6.06(3.78–9.73)	0	3.18(1.68-6.00)	0
Internal blood supply	7.70(4.99–11.88)	0	3.78(2.06–6.95)	0
Elasticity assessment		0		0
Soft	Reference		Reference	
Medium	3.24(1.96–5.35)	0	2.34(1.24–4.40)	0.009
Hard	16.48(9.58–28.36)	0	7.08(3.50-14.32)	0

### Development of the nomogram

Constructing a nomogram involves selecting covariates based on data availability, statistical significance, and clinical evidence rather than solely on statistical significance (*P* value) [14]. Although hyperechoic halo and orientation did not exhibit statistical significance in differentiating malignant from benign breast lesions in stepwise multivariate logistic regression analysis (*P* > 0.05), numerous previous studies and clinical experiences have indicated their strong association with breast cancer [17–20]. Therefore, they were still included in the multivariate logistic regression analysis (enter) to develop the prediction model.

**Fig. 1:**
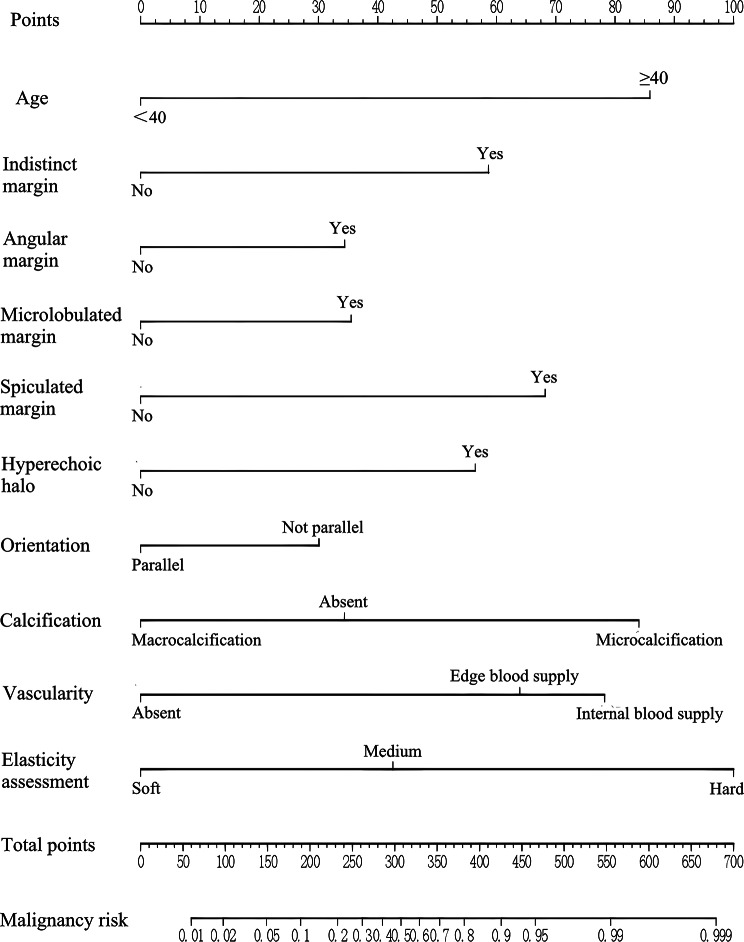
The nomogram for predicting the malignancy risk of breast lesions based on age and ultrasound characteristics. To use this nomogram, we first located the patient’s age and then drew a straight line up to the “Points” axis at the top to obtain the score associated with age. The process was repeated for each predictor. The scores of all predictors were added and the total score on the “Total points” axis was located. Finally, a line was drawn straight down to the “Malignancy risk” axis at the bottom to obtain the probability value

The final model included 10 predictors: age, indistinct margin, angular margin, microlobulated margin, spiculated margin, hyperechoic halo, orientation, calcification, vascularity, and elasticity assessment. These predictors were used to develop the prediction model and nomogram using multivariate logistic regression analysis (enter) (Table 6; Fig. 1).

**Table 6:** Multivariate logistic regression analysis (enter) in the training group

Predictors	OR (95% CI)	*P*
Age		
<40	Reference	
≥ 40	4.93(2.89–8.42)	0
Indistinct margin		
No	Reference	
Yes	2.97(1.75–5.05)	0
Angular margin		
No	Reference	
Yes	1.90(1.17–3.07)	0.01
Microlobulated margin		
No	Reference	
Yes	1.93(1.19–3.14)	0.008
Spiculated margin		
No	Reference	
Yes	3.55(1.69–7.46)	0.001
Hyperechoic halo		
No	Reference	
Yes	2.85(0.75–10.86)	0.125
Orientation		
Parallel	Reference	
Not parallel	1.75(0.93–3.29)	0.083
Calcification		0.001
Absent	Reference	
Macrocalcification	0.53(0.16–1.75)	0.296
Microcalcification	2.52(1.51–4.19)	0
Vascularity		0
Absent	Reference	
Edge blood supply	3.28(1.75–6.14)	0
Internal blood supply	4.28(2.37–7.71)	0
Elasticity assessment		0
Soft	Reference	
Medium	2.20(1.19–4.09)	0.012
Hard	6.41(3.24–12.66)	0

### Validation and performance of the nomogram

**Fig. 2:**
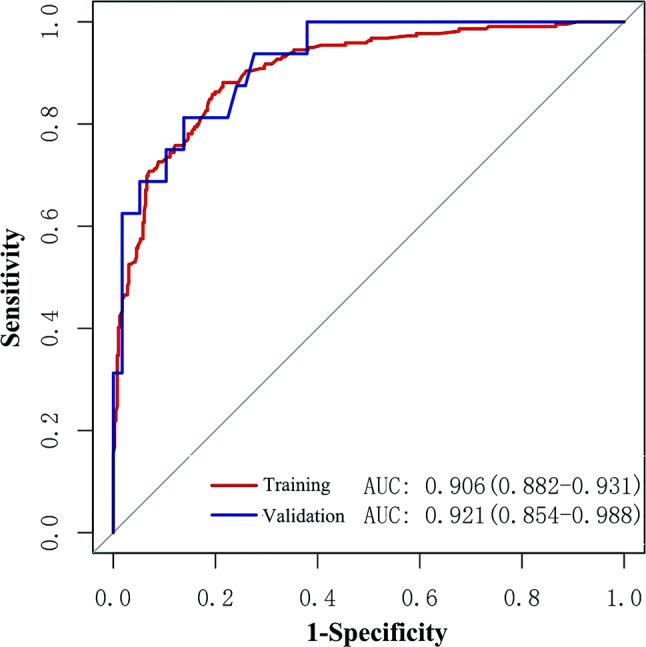
ROC curves of the nomogram, with an AUC of 0.906 in the training group and 0.921 in the validation group, demonstrating high diagnostic efficacy

**Fig. 3:**
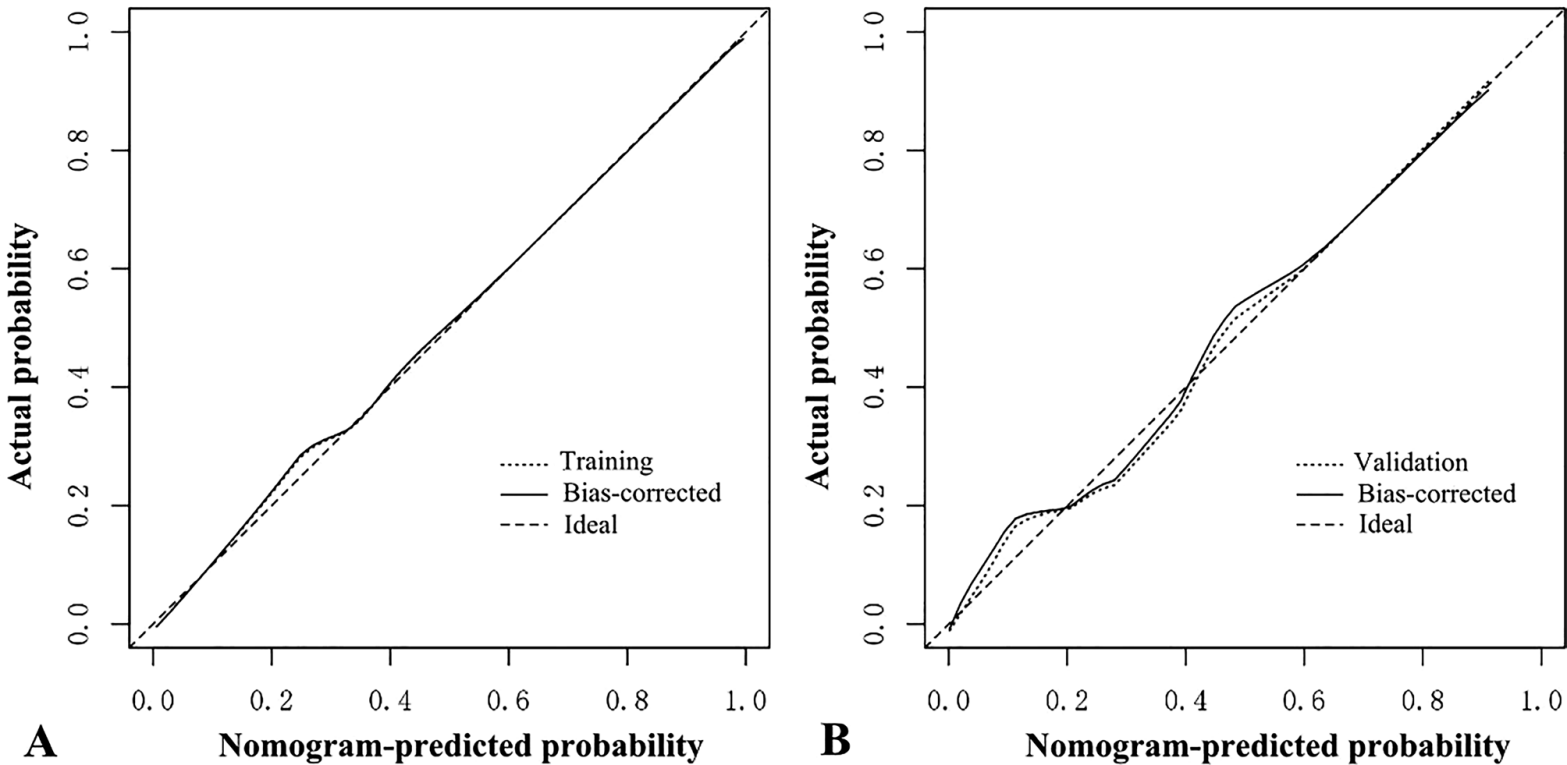
Calibration curves of the nomogram in the training (**A**) and validation groups (**B**), showing favorable agreement between the nomogram-predicted and actual risk of malignancy

**Fig. 4:**
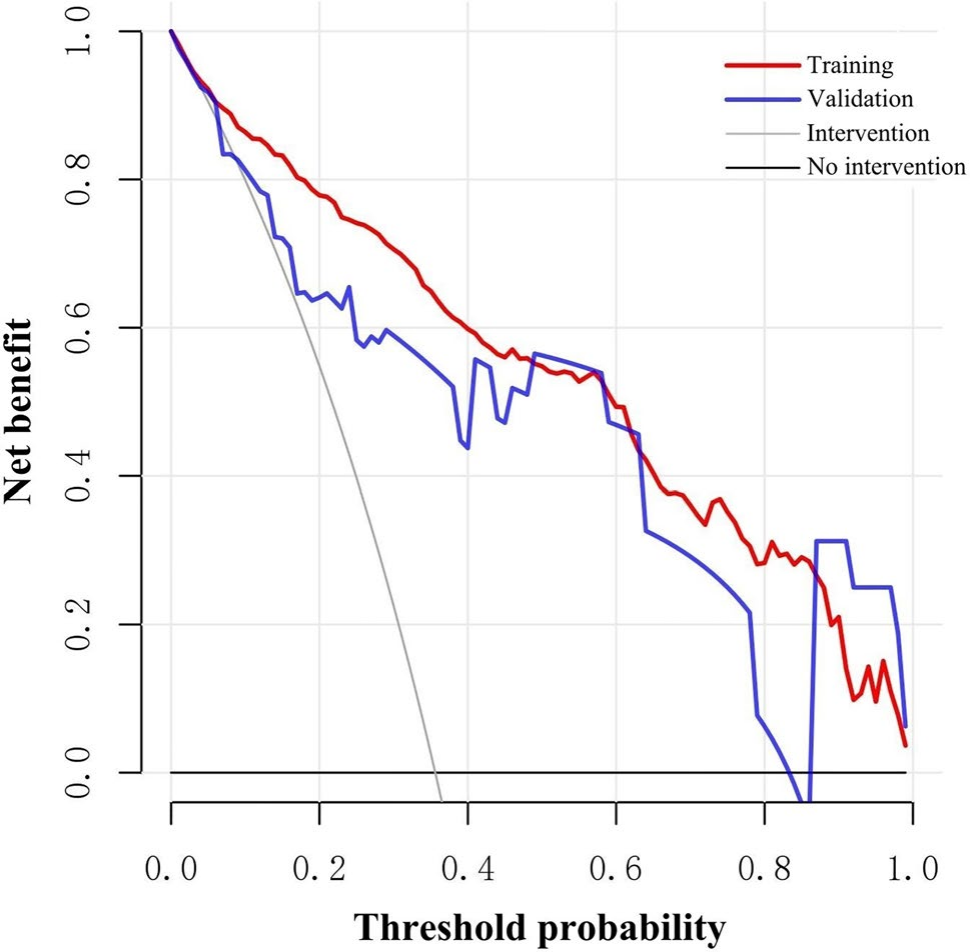
Decision curve analysis of the nomogram. The Y-axis “Net benefit” is calculated by weighing the harms of abandoning a biopsy against the adverse outcomes of performing an unnecessary biopsy. The X-axis “Threshold probability” represents the tendency to intervene. For example, when the threshold probability is set at 0.03, a lesion is classified as positive and requires biopsy if its probability predicted by the nomogram is ≥ 0.03; conversely, it is defined as negative and does not require biopsy if the predicted probability is < 0.03. “Intervention” indicates the net benefit when all patients are biopsied. “No intervention” indicates the net benefit when all the patients are not biopsied [21]

The ROC curves demonstrated the high diagnostic efficacy of the nomogram in both the training and validation groups (AUC = 0.906 and 0.921) (Fig. 2). Calibration curves displayed favorable agreement between the nomogram’s predicted malignancy risk of lesions and the actual malignancy risk in both groups (Fig. 3). Valuable threshold probabilities ranged from 0.03 to 1.00 in the training group and from 0.09 to 0.83 in the validation group. Within this threshold probability range, a net benefit can be achieved regardless of which threshold probability is adopted to guide biopsy decisions. Notably, the utility of deciding whether to biopsy or not based on the nomogram tended to diminish overall as the threshold probability increased (Fig. 4).

### Downgrading of BI-RADS category 4a lesions

**Fig. 5:**
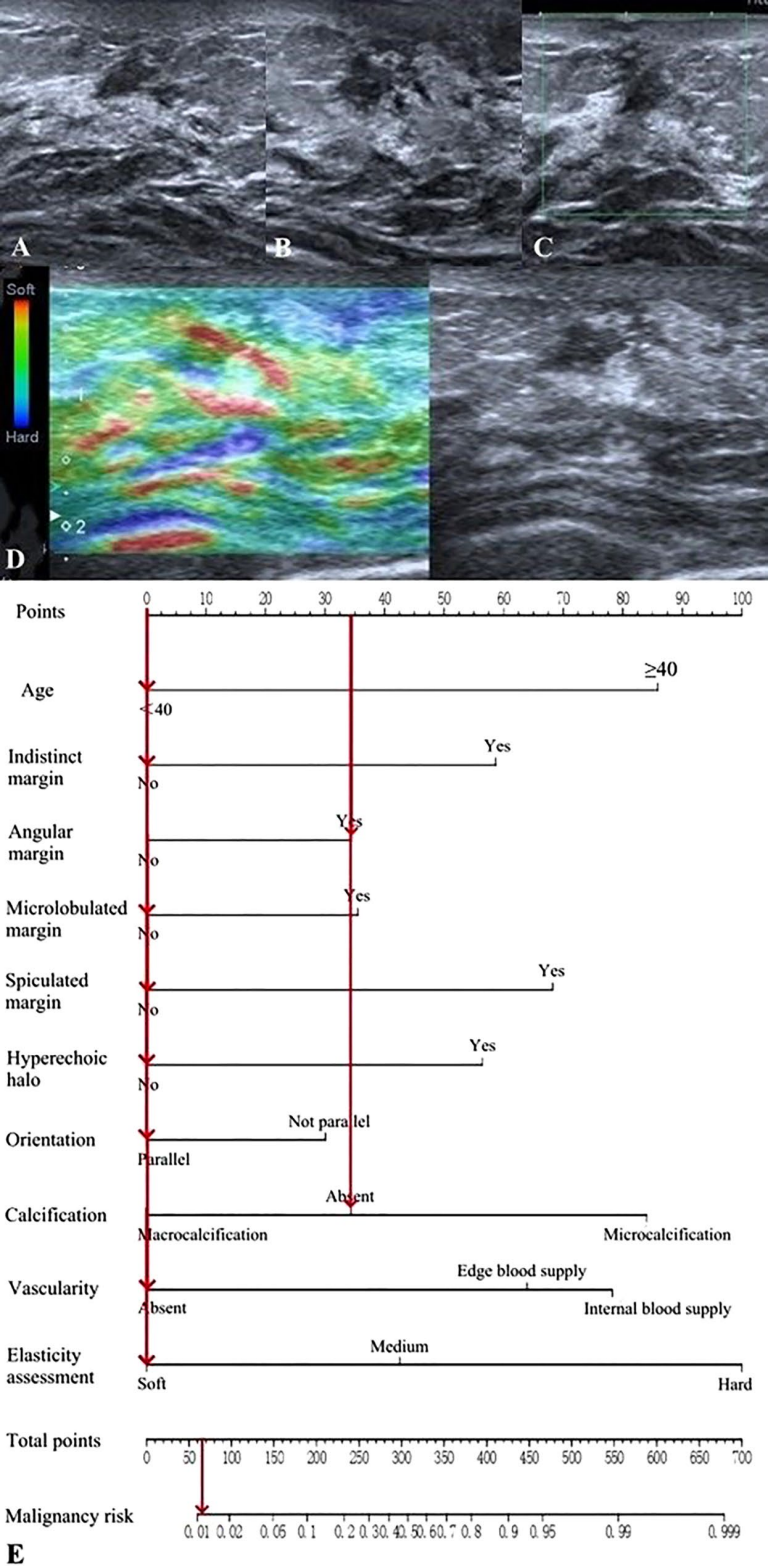
Images from a woman with a conventional BI-RADS 4a lesion and the pathology was benign. (**A**, **B**) Grayscale images (longitudinal and transverse planes): the lesion showed angular margin, parallel orientation, and absent calcification. (**C**) Color Doppler image: absent blood supply. (**D**) Elasticity assessment: soft. (**E**) The nomogram predicted a 0.012 probability of malignancy for this lesion

Conventional BI-RADS category 4a lesions were re-evaluated using the nomogram (Annexed Table 2). The lesions were downgraded to BI-RADS 3 if the nomogram predicted their probability of malignancy to be less than or equal to 2% (Fig. 5). In the training group, 13% (41/304) of conventional BI-RADS 4a lesions were downgraded by the nomogram, with only one malignancy (1/41, 2%). In the validation group, 17% (7/42) of conventional BI-RADS 4a lesions were downgraded through the nomogram, with no malignancies (0/7, 0%).

## Discussion

The escalating incidence of female breast cancer presents a significant health challenge [1, 22], underlining the importance of early detection to improve the treatment outcomes [2, 23]. Ultrasound stands as a cornerstone in breast cancer screening for Chinese women [4, 5]. However, its clinical application, guided by the ACR BI-RADS guideline, often relies on subjective examiner assessments, leading to an increasing risk of missed diagnosis and the misdiagnosis of breast cancer [10]. Furthermore, the broad range of malignancy probabilities (2%–95%) within BI-RADS categories which the ACR BI-RADS guideline recommends biopsy for, especially for BI-RADS 4a lesions, results in unnecessary biopsies, which strains healthcare resources and adds to patient anxiety [11].

To address these challenges, we developed a nomogram integrating conventional ultrasound and elastography. This nomogram aims to provide a more objective and accurate assessment of individual malignancy risks for breast lesions, and reduce unnecessary biopsies without increasing the risk of missed diagnosis.

According to the ACR BI-RADS guidelines, breast lesion margins are categorized as either circumscribed or not circumscribed, with the latter further classified into indistinct, angular, microlobulated, spiculated margins, and hyperechoic halo [6]. In our study, we used a detailed description of lesion margins, including circumscribed, indistinct, angular, microlobulated, spiculated margins, and hyperechoic halo. This approach offers a more comprehensive depiction than several other similar studies which often oversimplify margin descriptions as circumscribed or not circumscribed [24–27].

Multivariate logistic regression analysis confirmed age, margin characteristics, calcification, vascularity, and elasticity as independent predictors of breast cancer, consistent with existing literature [7, 28–30]. The significance of the echo pattern of lesions in relation to breast cancer is a subject of debate. While some studies have suggested that a hypoechoic pattern may indicate malignancy [31], others, including our own, have detected no statistical significance in using this pattern to differentiate between benign and malignant breast lesions [17].

Although hyperechoic halo and non-parallel orientation are often considered indicative of breast cancer [17, 18], they did not exhibit statistical significance in our analysis, which is likely due to their high specificity but low sensitivity and Youden index. Breast elastography includes strain elastography and shear-wave elastography. Strain elastography was adopted in this study due to its more frequent application at our institution. It remains to be validated whether the stiffness assessments of breast lesions obtained by shear-wave elastography corresponds to the “soft”, “medium” and “hard” classifications in our nomogram. Therefore, the nomogram may not be directly applicable to shear-wave elastography-based assessments. Stiffness assessment is directly correlated with the histological composition of lesions. Increased stiffness is generally associated with a higher probability of malignancy [32]. However, some malignant tumors with special pathological features (e.g., mucinous carcinoma, intratumoral necrosis/cystic degeneration, early invasive carcinoma, etc.) may appear softer on SE. In such cases, when confronting a lesion with suspicious morphological features but soft elastographic presentation, careful BI‑RADS classification is imperative, and soft elastographic findings alone should not warrant a downgrade in category. These exceptions underscore the importance of considering multiple factors in a comprehensive risk assessment model like our nomogram.

The nomogram developed in our study demonstrated superior performance than most other nomograms developed for assessing the malignancy risk of breast lesions [24, 33–36]. The calibration curves showed favorable agreement between the nomogram-predicted malignancy risk and the actual risk, ensuring the reproducibility and reliability of the nomogram. In addition, DCA was used to assess the clinical utility of the nomogram. DCA, first proposed by Vickers and Elkin in 2006 [37], simultaneously considers discrimination and calibration of the nomogram, integrates decision-makers and patients’ preferences, weighs the risk of missed diagnosis against the risk of misdiagnosis and clearly considers the clinical consequences of the decisions to fulfill the actual needs of clinical decision making [21, 38]. DCA indicated that our nomogram had better net benefits over a wide range of threshold probabilities, suggesting good clinical applicability and potential to optimize the ACR BI-RADS guidelines. DCA can evaluate the clinical utility of the nomogram in a manner that aligns more comprehensively with clinical practice. However, only a few published studies similar to ours have conducted DCA on nomograms [26, 27, 34]. Performing DCA on our nomogram was a strength of our study, yielding favorable results.

In our study, the nomogram was used to re-categorize conventional BI-RADS 4a lesions, resulting in the downgrade of some lesions to BI-RADS 3, with a missed diagnosis risk consistent with the likelihood of malignant for BI-RADS category 3 as specified in the ACR BI-RADS guideline. This indicates that the nomogram has the potential to downgrade BI-RADS 4a lesions to BI-RADS 3, thereby avoiding unnecessary biopsies without increasing the risk of missed diagnosis, which is consistent with the findings from other similar studies [24, 33, 35]. Among these downgraded lesions, one malignant lesion in the training group was missed, resulting in a 2% missed diagnosis rate. Although this rate falls within the likelihood of malignant for BI-RADS category 3 as defined by the ACR BI-RADS guideline [6], such misclassification may delay the timing of treatments such as surgery, radiotherapy, and chemotherapy, thereby increasing the healthcare costs and difficulty of subsequent treatment for patients. The ACR BI-RADS guideline recommends a short-interval(6-month) follow-up or continued surveillance(12-month) for BI-RADS category 3 lesions [6]. For lesions empirically classified as BI-RADS 4a but downgraded to BI-RADS 3 by the nomogram, shortening the follow-up interval to approximately 3 months is beneficial for mitigating the adverse clinical consequences caused by potential misclassification.

Contrast-enhanced ultrasonography (CEUS) significantly improves the diagnostic accuracy for breast lesions, particularly for indeterminate BI-RADS category 3–4 lesions [39]. However, given its high requirements for ultrasound equipment and operators, along with the risk of contrast agent allergy, CEUS is not the preferred method for routine screening or follow-up of breast lesions and thus was not included in this study.

This study and the model have several limitations. First, its single-center retrospective design introduces a certain degree of selection bias. Although internal validation with bootstrap resampling method and external validation with an independent cohort collected from the same center but during a different time period was performed, the small validation sample and lack of multicenter external validation still pose a risk of overfitting and limit the model’s generalizability. Second, constrained by the retrospective data collection approach, only age was included as a clinical variable, while other critical clinical factors closely associated with breast cancer (e.g., family history of breast cancer and hormone therapy history) were not incorporated. This deficiency restricts the model’s comprehensiveness. Third, the nomogram depends on ultrasound characteristics subjectively interpreted by examiners. Inter-observer variability in ultrasound interpretation may therefore compromise the reliability and reproducibility of the model. Finally, the absence of a web-based calculator necessitates manual calculation of risk probabilities, which hinders the clinical utility of the nomogram. To address these limitations, future studies should aim to incorporate more comprehensive clinical data, conduct larger prospective multicenter validations, and develop user-friendly online tools to facilitate the clinical application of the nomogram.

## Conclusion

In conclusion, we developed a nomogram based on conventional ultrasound and elastography that shows promise in predicting the malignancy risk for BI-RADS category 3–5 lesions. Despite its limitations, the nomogram offers a valuable tool for improving the accuracy of breast lesion assessment and guiding clinical decision-making. Further research is warranted to address the limitations and validate the nomogram’s performance in diverse populations. 

## Electronic Supplementary Material

Below is the link to the electronic supplementary material.

Supplementary Material 1

Supplementary Material 2

## Data Availability

The raw data contains private information of the patients, so we are sorry that we do not wish to share all the raw data. As an alternative, we have provided a summary of the data in a supplementary file. Data is provided within the manuscript and supplementary information files.
