# Supplementary material for: A nomogram based on conventional ultrasound and elastography for diagnosing BI-RADS category 3–5 lesions
Source: BMC Med Imaging. 2026 Feb 11;26:138. doi: 10.1186/s12880-026-02216-3 (PMC12998031; doi:10.1186/s12880-026-02216-3)
Supplement: Supplementary file 2 — Supplementary Material 2 [file 12880_2026_2216_MOESM2_ESM.docx]

**Appendix**

Annexed Table 1 Multicollinearity test for predictors in the training group

| Predictors | Tolerance | Variance inflation factors |
| --- | --- | --- |
| Age | 0.953 | 1.049 |
| Shape | 0.435 | 2.297 |
| Circumscribed margin | 0.296 | 3.376 |
| Indistinct margin | 0.633 | 1.579 |
| Angular margin | 0.604 | 1.655 |
| Microlobulated margin | 0.66 | 1.515 |
| Spiculated margin | 0.789 | 1.268 |
| Hyperechoic halo | 0.703 | 1.423 |
| Orientation | 0.878 | 1.139 |
| Posterior features | 0.875 | 1.143 |
| Calcification | 0.902 | 1.109 |
| Vascularity | 0.869 | 1.151 |
| Elasticity assessment | 0.791 | 1.265 |

Annexed Table 2 Re-evaluation of conventional BI-RADS 4a lesions by the nomogram

|  | | Conventional BI-RADS 4a | |
| --- | --- | --- | --- |
|  |  | Training group | validation group |
| Nomogram | BI-RADS 3 | 41 | 7 |
|  | BI-RADS 4a | 123 | 17 |
|  | BI-RADS 4b | 112 | 18 |
|  | BI-RADS 4c | 25 | 0 |
|  | BI-RADS 5 | 3 | 0 |
|  | Total | 304 | 42 |
